# Inflammatory biomarkers of neutrophil-to-lymphocyte and platelet-to-lymphocyte ratios in 563 severe OSA patients before and after surgery

**DOI:** 10.1186/s40463-023-00653-6

**Published:** 2023-07-27

**Authors:** Chung-Wei Lin, Pei-Wen Lin, Li-Wen Chiu, Han-Tan Chai, Chun-Tuan Chang, Michael Friedman, Anna M. Salapatas, Sara Rahavi-Ezabadi, Hsin-Ching Lin

**Affiliations:** 1 Department of Education, Kaohsiung Chang Gung Memorial Hospital, Kaohsiung, Taiwan; 2 Division of Glaucoma, Department of Ophthalmology, Kaohsiung Chang Gung Memorial Hospital, Kaohsiung, Taiwan; 3 College of Medicine, Chang Gung University, Taoyuan, Taiwan; 4 Sleep Center, Kaohsiung Chang Gung Memorial Hospital, Kaohsiung, Taiwan; 5 Division of Cardiology, Department of Internal Medicine, Kaohsiung Chang Gung Memorial Hospital, Kaohsiung, Taiwan; 6 Department of Business Management, Institute of Healthcare Management, Institute of Biomedical Sciences, National Sun Yat-Sen University, Kaohsiung, Taiwan; 7 Department of Otolaryngology, Division of Sleep Surgery, Rush University Medical Center, Chicago, IL USA; 8 Department of Otolaryngology, Advanced Center for Specialty Care, Advocate Illinois Masonic Medical Center, Chicago, IL USA; 9 Otorhinolaryngology Head and Neck Surgery Department, Otorhinolaryngology Research Center, Children’s Medical Center, Tehran University of Medical Sciences, Tehran, Iran; 10 Department of Otolaryngology, Robotic Surgery Center and Center for Quality Management, Kaohsiung Chang Gung Memorial Hospital, 123, Ta-Pei Rd., Niao-Sung District, Kaohsiung, 833 Taiwan

**Keywords:** Snoring, Obstructive sleep apnea, Inflammatory biomarker, Coronary artery disease, Stroke

## Abstract

**Background:**

Evidence has proved that high neutrophil-to-lymphocyte ratio (NLR) and platelet-to-lymphocyte ratio (PLR) were risk factors for cardiovascular comorbidities. The alterations of NLR and PLR following obstructive sleep apnea (OSA) treatment were under studied and thus should be investigated. This study aimed to evaluate the changes of inflammatory biomarkers including NLR and PLR in severe OSA patients after surgical interventions of the upper airway, and their relationships with improvements in polysomnographic (PSG) parameters.

**Methods:**

This retrospective cohort study included 563 consecutive severe OSA patients at a tertiary academic medical center who received OSA surgery, as well as underwent pre- and post-operative polysomnographic (PSG) examinations and blood tests. The changes of major PSG estimates, NLR, and PLR before and at least 3 months after OSA surgery were analyzed using paired t-tests with subgroup analyses. Pearson’s correlations were performed to discover which PSG parameter contributed to the improvement of the values.

**Results:**

After OSA surgery, the major PSG estimates, NLR and PLR dropped significantly in the overall population. In those with a higher preoperative NLR (pre-operative NLR≧3) and PLR (pre-operative PLR≧150), the mean (SD) difference of NLR (− 0.8 [1.6], 95% CI − 1.5 to − 0.2) and PLR (− 41.6 [40], 95% CI − 52.8 to − 30.5) were even more substantial. The changes of the “apnea, longest (*r* = 0.298, *P* = .037)” and “hypopnea, longest (*r* = 0.321, *P* = .026)” were found significantly related to the improvement of PLR.

**Conclusion:**

NLR and PLR did significantly drop in severe OSA patients following OSA surgery, and this could be related to the alterations of sleep indices. The findings could possess clinical importance for severe OSA patients after OSA surgeries in reducing possible OSA-associated cardiovascular comorbidities.

## Background

Obstructive sleep apnea (OSA) has been considered as a major health concern worldwide and proved to be highly associated with cardiovascular diseases, cerebrovascular morbidities, cognition impairment, and even higher mortality rate, which deserves close attention by clinicians [1, 2]. Due to the high global prevalence of OSA, ranging from 9% to as high as 38% [3], to detect the OSA-related comorbidities at early stages and follow up the changes of possible treatment outcome-associated cardiovascular biomarkers are thus crucial. Clinical consensus also highlights the need of biomarkers to guide the treatment decision-making and evaluate the outcomes in depth.

For those symptomatic patients with OSA in need of treatments, methods such as continuous positive airway pressure (CPAP) and oral appliance are usually the first-line therapy [4, 5]. Furthermore, those who failed to be compliant with the conservative OSA therapy, surgical treatments of the upper airway usually serve as salvage managements for treating the disease; these often include a multilevel correction of the anatomical structures from the nasal to hypopharyngeal regions [6, 7]. Patients undergoing OSA surgery may gain improvement in not only sleep, but also some indicators of other systems such as blood pressure [8], metabolic profiles [9], brain functions [10], and even ophthalmic microstructures [11].

Recently, there have been emerging evidences promoting that certain biomarkers may also be highly associated with OSA. Neutrophil-to-lymphocyte ratio (NLR) and platelet-to-lymphocyte ratio (PLR), two novel biomarkers reflecting the endogenous stress and risks to develop various diseases [12, 13], have been studied in some of the OSA-related studies [14–16]. Since NLR and PLR also possess the potential to determine the inflammation status and stress extent a person experienced [12, 13], decreases in both of the two parameters after OSA treatments are expected, which has been previously explored in OSA patients treated with conservative therapies such as CPAP and mandibular advancement device (MAD) [17–19]. However, many OSA patients could not tolerate conservative treatments, and will select OSA surgeries as alternatives. Therefore, we conducted this first study to investigate the NLR and PLR changes following surgical interventions of the upper airway in severe OSA patients, as well as their relationships with changes of the estimates in polysomnography (PSG) after the treatment.

## Methods

This retrospective cohort study was approved by the Institutional Review Board (IRB) of Chang Gung Memorial Hospital (CGMH) Ethical Committee (CGMH IRB No. 202200848B0) and followed the Declaration of Helsinki.

### Study design

This study enrolled patients with severe OSA (apnea/hypopnea index/AHI more than 30/hr.) and analyzed their NLR and PLR changes before and after OSA surgery, as well as in the subgroup analyses based on the preoperative NLR and PLR values. Morning venous blood samples were collected at 8–9 a.m. to exclude the possible influence of circadian variations. The hospital’s central laboratory measured the levels of complete blood count and differential count; and this study majorly focused on the levels of neutrophil, lymphocyte, and platelet.

The cutoff value of NLR for grouping was 3, based on the common scale to determine whether a person was under excessive stress [20]; and the cutoff value of PLR for grouping was defined as 150, since most studies took patients with PLR higher than this level as high-risk ones to develop multiple diseases [21–23]. Additionally, the alterations of the major PSG estimates and their correlations with changes of the NLR and PLR were also investigated.

### Patient selection

Patients with severe OSA, who had failed non-surgical OSA treatments (e.g. CPAP, oral appliance) and then received OSA surgery as the alternative management from June, 2006 to March, 2022, were recruited. Patients with chronic usage of multiple medications known to affect sleep or those with heavy alcohol use or smoking, intractable hypertension, severe coronary or cerebral vascular disease, active infection status, history of psychosis or autoimmune disorders, or incomplete pre-operative and post-operative data were excluded. All OSA surgeries were performed via general anesthesia by the author (H-C Lin). Multilevel surgery including palatal surgery, tongue base surgery, and nasal surgery (if necessary) was performed. The more detailed surgical techniques used in this cohort are described as our previous literature [24–27]. All of the enrolled patients had full-night PSG examinations and blood tests (complete blood cell count and differential count) before being treated, and at least three months after the surgical intervention for OSA.

### Data collection

We retrospectively collected the following data of the patients by reviewing the medical records: age, gender, Epworth Sleepiness Scale (ESS), body mass index (BMI, kg/m^2^) and major PSG-related indices, such as sleep efficiency (%), percentage of sleep stages on N1, N2, N3, and rapid eye movement (REM), AHI (/hr.), AHI in REM (/hr.), longest apnea period (apnea, longest; sec.), longest hypopnea period (hypopnea, longest; sec.), percentage of oxygen saturation less than 90% (PO_2_ < 90%), mean saturation of oxygen (mO_2_, %), lowest saturation of oxygen (LSAT, %), oxygen desaturation index (ODI, /hr.), arousal index (/hr.), and snoring index (/hr.). In addition, blood parameters including neutrophil count (×1000/μL), platelet count (×1000/μL), and lymphocyte count (×1000/μL) were recorded for the calculation of NLR and PLR for analyses.

### Statistical analysis

The changes of the major PSG estimates and the alterations of NLR and PLR before and after OSA surgery were analyzed using paired t-tests. Subgroup analyses based on NLR (< 3 or ≧ 3) and PLR (< 150 or ≧ 150) with paired t-tests were also compared and analyzed. Moreover, the associations among the changes of the major PSG parameters and alterations of NLR (in the group of preoperative NLR ≧ 3) and PLR (in the group of preoperative PLR ≧ 150) were investigated via Pearson’s correlation analysis. Significance was accepted when the two-tailed *P* < 0.05. All statistical analyses were performed using SPSS software (version 22.0, Chicago, IL, USA).

## Results

In total, 563 patients with severe OSA were enrolled for the final study. The basic features and the PSG characteristics before and after surgery were presented as Table 1. All of the measured PSG estimates, including ESS, BMI (kg/m2), sleep efficiency (%), N1 (%), N2 (%), N3 (%), REM (%), AHI (/hr.), AHI in REM (/hr.), apnea, longest (sec.), hypopnea, longest (sec.), PO2 < 90% (%), mO2 (%), LSAT (%), ODI (/hr.), arousal index (/hr.), and snoring index (/hr.) improved significantly.

**Table 1:** Changes of major polysomnographic parameters in the 563 severe OSA patients before and after surgery

Characteristics	Mean (SD)			95% CI for difference	*P* value^a^
	Pre-operative	Post-operative	Difference		
ESS	9.7 (4.7)	7.8 (4.2)	− 2.0 (4.6)	− 2.4 to − 1.6	< 0.001
BMI (kg/m^2^)	27.4 (3.3)	26.5 (3.2)	− 0.9 (1.7)	− 1.1 to − 0.8	< 0.001
Sleep efficiency (%)	83.7 (13.0)	86.2 (12.6)	2.5 (15.1)	1.2 to 3.9	< 0.001
N1 (%)	55.9 (20.9)	41.2 (21.6)	− 14.6 (25.4)	− 16.9 to − 12.3	< 0.001
N2 (%)	27.4 (18.4)	38.3 (18.1)	10.9 (21.6)	8.9 to 12.8	< 0.001
N3 (%)	1.4 (3.9)	2.1 (4.4)	0.6 (5.0)	0.2 to 1.1	0.006
REM (%)	15.0 (7.0)	18.2 (6.9)	3.2 (8.6)	2.4 to 4.1	< 0.001
AHI (/hr.)	57.6 (18.1)	34.9 (31.5)	− 22.8 (32.9)	− 25.7 to − 19.8	< 0.001
AHI in REM (/hr.)	55.6 (38.6)	35.3 (24.8)	− 20.3 (41.0)	− 24.0 to − 16.6	< 0.001
Apnea, longest (sec.)	59.0 (22.2)	45.6 (33.4)	− 13.3 (32.2)	− 16.3 to − 10.3	< 0.001
Hypopnea, longest (sec.)	67.3 (30.6)	81.6 (42.6)	14.2 (49.9)	9.6 to 18.9	< 0.001
PO2 < 90% (%)	16.4 (17.1)	8.4 (13.7)	− 8.0 (15.1)	− 9.4 to − 6.6	< 0.001
mO2 (%)	93.6 (3.2)	94.9 (2.2)	1.3 (2.9)	1.1 to 1.6	< 0.001
LSAT (%)	71.6 (12.2)	79.5 (10.8)	7.9 (11.5)	6.8 to 8.9	< 0.001
ODI (/hr.)	46.3 (22.1)	24.3 (22.3)	− 22.0 (23.2)	− 24.1 to − 19.9	< 0.001
Arousal index (/hr.)	47.7 (26.7)	32.3 (24.7)	− 15.4 (31.3)	− 18.3 to − 12.5	< 0.001
Snoring index (/hr.)	394.2 (206.0)	301.4 (224.0)	− 92.8 (272.3)	− 117.5 to − 68.1	< 0.001

OSA: obstructive sleep apnea/hypopnea syndrome; ESS: Epworth Sleepiness Scale; BMI: body mass index; REM: rapid eye movement; AHI: apnea/hypopnea index; PO_2_ < 90%: percentage of time with saturation of oxygen below 90%; mO_2_: mean saturation of oxygen; LSAT: lowest saturation of oxygen; ODI: oxygen desaturation index

^a^Statistical analyses were performed using *paired t-tests*, and *P* < *.05* was viewed as of significance

As to the alterations of NLR and PLR before and after OSA surgery (Table 2), the paired t-tests revealed that in the overall population, both NLR (mean [SD] difference, − 0.1 [0.8], 95% CI − 0.2 to 0.0, *P* = 0.004) and PLR (mean [SD] difference, − 9.6 [30.3], 95% CI − 13.0 to − 6.2, *P* < 0.001) decreased significantly. The subgroup analysis of the pre-operative NLR disclosed that the decline of NLR level was statistically significant only in those with the NLR values higher than 3 (mean [SD] difference, − 0.8 [1.6], 95% CI − 1.5 to − 0.2, *P* = 0.013), but not in those lower than 3 (mean [SD] difference, − 0.1 [0.6], 95% CI − 0.1 to 0.0, *P* = 0.082). On the other hand, in the subgroup analysis based on the pre-operative PLR, the decrease amount of PLR after surgery were statistically substantial in both the lower PLR subgroup (mean [SD] difference, − 3.1 [23.2], 95% CI − 5.9 to − 0.2, *P* = 0.033) and higher PLR subgroup (mean [SD] difference, − 41.6 [40.0], 95% CI − 52.8 to − 30.5, *P* < 0.001); the latter demonstrated an even greater extent of PLR decrease following the surgical intervention.

**Table 2:** Changes of the NLR and PLR before and after OSA surgery with subgroup analyses based on the pre-operative NLR and PLR values

	Mean (SD)			95% CI for difference	*P* value^a^
	Pre-operative	Post-operative	Difference		
NLR					
Overall	1.9 (0.7)	1.7 (0.8)	− 0.1 (0.8)	− 0.2 to 0.0	0.004
Pre-operative NLR < 3 (n = 525)	1.7 (0.5)	1.6 (0.6)	− 0.1 (0.6)	− 0.1 to 0.0	0.082
Pre-operative NLR≧3 (n = 38)	3.6 (0.6)	2.7 (1.8)	− 0.8 (1.6)	− 1.5 to − 0.2	0.013
PLR					
Overall	118.3 (38.9)	108.7 (34.0)	− 9.6 (30.3)	− 13.0 to − 6.2	< 0.001
Pre-operative PLR < 150 (n = 481)	104.8 (24.1)	101.7 (27.1)	− 3.1 (23.2)	− 5.9 to − 0.2	0.033
Pre-operative PLR≧150 (n = 82)	184.8 (28.4)	143.2 (42.9)	− 41.6 (40.0)	− 52.8 to − 30.5	< 0.001

OSA: obstructive sleep apnea/hypopnea syndrome; NLR: neutrophil-to-lymphocyte ratio; PLR: platelet-to-lymphocyte ratio

^a^Statistical analysis were performed using *paired t-tests*, and *P* < *.05* was viewed as of significance

The Pearson’s correlation was performed to further identify the relationships among the changes in the various PSG parameters, NLR, and PLR. In Table 3, which was based on the subgroup of “pre-operative NLR higher than 3”, none of the alterations of the measured PSG parameters were correlated with the changes of NLR. Nevertheless, in the subgroup of “pre-operative PLR higher than 150” (Table 4), two parameters including the decrease of “apnea, longest (*r* = 0.298, *P* = 0.037)” and “hypopnea, longest (*r* = 0.321, *P* = 0.026)” were significantly positively associated with the improvement of PLR.

**Table 3:** Pearson correlations between changes of the NLR and changes of the major polysomnographic parameters in the *higher pre-operative NLR group* (pre-operative NLR≧3)

	ΔAHI	ΔREM	ΔApnea, longest	ΔHypopnea, longest	Δ% of mO_2_ < 90%	ΔMean O_2_%	ΔLSAT	ΔODI	ΔArousal index
ΔNLR									
* r*	0.268	0.306	0.249	− 0.086	− 0.023	0.069	− 0.055	0.253	0.067
* P* value^a^	0.196	0.137	0.252	0.697	0.923	0.749	0.795	0.232	0.762

NLR, neutrophil-to-lymphocyte ratio; Δ: changes of the data before and after treatment; AHI, apnea/hypopnea index; REM, rapid eye movement; mO2: mean saturation of oxygen; LSAT, lowest oxygen saturation; ODI: oxygen desaturation index

^a^Statistical significance was accepted when *P* < *.05*

**Table 4:** Pearson correlations between changes of the PLR and changes of the major polysomnographic parameters in the *higher pre-operative PLR group* (pre-operative PLR≧150)

	ΔAHI	ΔREM	ΔApnea, longest	ΔHypopnea, longest	Δ% of mO_2_ < 90%	ΔMean O_2_%	ΔLSAT	ΔODI	ΔArousal index
ΔPLR									
* r*	0.153	0.201	0.298	0.321	− 0.076	0.077	− 0.096	0.139	0.130
* P* value^a^	0.278	0.153	0.037	0.026	0.606	0.595	0.498	0.336	0.374

PLR, platelet-to-lymphocyte ratio; Δ: changes of the data before and after treatment; AHI, apnea/hypopnea index; REM, rapid eye movement; mO2: mean saturation of oxygen; LSAT, lowest oxygen saturation; ODI: oxygen desaturation index

^a^Statistical significance was accepted when *P* < *.05*

## Discussion

To the best of our knowledge, this is the first study investigating the NLR and PLR changes following surgical treatments in patients with severe OSA. The results demonstrated that both NLR and PLR declined significantly, and greater amount of decreases in both of the values were observed in those with higher pre-operative NLR and PLR, respectively. Moreover, the changes of “apnea, longest” and “hypopnea, longest” after OSA surgery were associated with the change of PLR.

Regarding the possible pathophysiology, it has been recognized that the immune response activated by intermittent hypoxia probably contributes to the rising inflammatory biomarkers in OSA patients, which attributes to the activation of nuclear factor-κB (NF‑κB) pathways [28, 29]. During the process, neutrophils and lymphocytes are the main roles participating in the reaction; NLR, furthermore, serves as a more effective and comprehensive measurement in reflecting the stress burden, since the ratio includes two distinct immune pathways compared to the single leukocyte parameter [14]. Platelet, the key factor for tissue repair, is also an important participant involved in the immune response; it aggregates to each other more than usual, and is associated with a higher cytokine production during the period [30]. The high level of circulating platelet-lymphocyte complex can be an indicator of platelet activation, and thus make PLR valuable regarding the inflammation process in OSA patients [30].

Previous studies have also surveyed the relationships of NLR and PLR in OSA patients through observational longitudinal studies. For example, regarding NLR, a multicenter study by Altintas et al. [31] stated that NLR was significantly higher in those with severe OSA, which was similarly claimed by Sunbul et al. [32] and Oyama et al. [33]; considering PLR, a study encompassing 424 patients by Koseoglu et al. [16] also promoted that PLR was strongly associated with OSA severity. A meta-analysis comprising 2,259 OSA patients, which investigated the association between NLR and OSA, further proved that NLR was significantly higher in patients with OSA than in controls [14]. Given that NLR and PLR have been proved to be evident indicators for cardiovascular comorbidities [12, 13], the above-mentioned evidence indicated that the administration of NLR and PLR could help not only assess high-risk OSA patients with clinical values, but also further evaluate the risks for certain probable OSA-related diseases.

According to our results, the majority of PSG estimates improved substantially after OSA surgeries, so did NLR and PLR. It was also noteworthy that “hypopnea, longest” increased after surgery, which might be due to the human physiology with inevitable shifting of apnea period to a “relatively less severe” hypopnea period, and thus making the overall post-surgical ODI level dropped significantly as well. Additionally, those with higher pre-operative NLR and PLR values experienced greater decreases in the level of the parameters. Therefore, lower risks for cardiovascular comorbidities might be expected among these groups of treated patients. However, despite the changes in both “apnea, longest” and “hypopnea, longest” were able to explain the improvement of PLR, no PSG parameters were found correlating to the decrease of NLR. Given that the immune response and oxidative stress resulting from OSA may be attenuated and improved following OSA treatments [34], which are due to the shortening of the longest apnea and hypopnea period, it is unsurprising that the post-operative PLR is lower compared to the preoperative values. Still, the insignificant results among the PSG parameters in the analysis of NLR implies that there might still be other unmeasured factors contributing to the decreases, which needs to be further discovered.

In the past, there were limited studies focusing on the influence of OSA treatments on changes of NLR and PLR, which were primarily based on non-invasive therapies. The results were inconsistent, but decreases in these two parameters were mostly seen. For instance, there were studies exploring the effect of CPAP and MAD on the decline of NLR, and the results turned out that NLR were indeed significantly lower than the pre-treatment values [17, 18]. On the other hand, a study [19] inspecting the NLR and PLR changes following CPAP treatment revealed that both of the parameters did not decrease remarkably; despite the insignificant results, the authors also clarified that the exclusion of comorbid patients may be the reason. Based on our study, the results may support the discussion of the post-treatment decline of these two values, as well as their potential connections to the development of certain comorbidities based on a relatively larger population. In addition, this first study evaluating the effect of OSA surgery on the improvement of NLR and PLR may provide potential insights for OSA patients unable to adhere to conservative OSA treatments in the future.

There are some limitations of the study. First, the enrolled-populations are all severe OSA patients. It is unknown whether the results of the study may be altered in those with less severe OSA. Second, the types of OSA surgery were not differentiated. Since upper airway modifications of OSA are all salvage treatments secondary to the conservative therapies, and all are based on the corrections of abnormal anatomical structures pre- and peri-operatively, the bias may be thus minimized regardless of the classifications of the OSA surgeries. Third, the improvement of NLR and PLR following OSA treatment might still possibly be affected by other clinical factors, such as intake status, which are unmeasured in this study. Lastly, the study was retrospective and lacked a control group. Future studies are warranted to figure out the impact of various factors on NLR and PLR to better understand this issue with a higher level of evidence.

## Conclusion

OSA surgery can significantly improve the majority of PSG parameters, NLR, and PLR. The changes of the longest period of apnea and hypopnea are the potential clinical factors associated with the improvement of PLR. More future studies are still needed to elucidate this topic in the field of sleep-related breathing disorders in depth.

## Data Availability

The datasets used and/or analysed during the current study are available from the corresponding author on reasonable request.
